# Photoselective Protective Netting Improves “Honeycrisp” Fruit Quality

**DOI:** 10.3390/plants9121708

**Published:** 2020-12-04

**Authors:** Sara Serra, Stefano Borghi, Giverson Mupambi, Hector Camargo-Alvarez, Desmond Layne, Tory Schmidt, Lee Kalcsits, Stefano Musacchi

**Affiliations:** 1 Tree Fruit Research and Extension Center, Washington State University, Wenatchee, WA 98801, USA; stefano.borghi@wsu.edu (S.B.); gmupambi@umass.edu (G.M.); hac809@student.bham.ac.uk (H.C.-A.); lee.kalcsits@wsu.edu (L.K.); stefano.musacchi@wsu.edu (S.M.); 2 Department of Horticulture, Washington State University, Pullman, WA 99164, USA; drl0021@auburn.edu; 3 Currently, Department of Horticulture, Auburn University, Auburn, AL 36849, USA; 4 Washington Tree Fruit Research Commission (WTFRC), Washington State University, Wenatchee, WA 98801, USA; Tory@treefruitresearch.com

**Keywords:** solar radiation, scattered light, spectroradiometer, *Malus domestica* Borkh., sunburn, bitter pit

## Abstract

High temperatures, wind, and excessive sunlight can negatively impact yield and fruit quality in semi-arid apple production regions. Netting was originally designed for hail protection, but it can modify the light spectrum and affect fruit quality. Here, pearl, blue, and red photoselective netting (≈20% shading factor) was installed in 2015 over a commercial “Cameron Select^®^ Honeycrisp” orchard. Our research objectives were to (1) describe the light quantity and quality under the colored nets compared to an uncovered control and (2) investigate the effect of Photoselective nets on “Honeycrisp” apple quality for two growing seasons. Light transmittance and scattering for each treatment were measured with a spectroradiometer, and samples for fruit quality analyses were collected at harvest. PAR (photosynthetic active radiation), UV, blue, red, and far-red light were lower underneath all netting treatments compared to an uncovered control. The scattered light was higher under the pearl net compared to other colors, while red and far-red light were lower under the blue net. For two consecutive years, trees grown under the photoselective nets intercepted more incoming light than the uncovered trees with no differences among the three colors. In both years, trees under red and blue nets had more sunburn-free (clean) apples than pearl and control. Red color development for fruit was lower when nets were used. Interestingly, bitter pit incidence was lower underneath red nets for both years. Other than red color development, “Honeycrisp” fruit quality was not appreciably affected by the use of netting. These results highlight the beneficial effect of nets in improving light quality in orchards and mitigating physiological disorders such as bitter pit in “Honeycrisp” apple.

## 1. Introduction

Apple production regions such as Washington State that are characterized as a semi-arid climate [1,2] may benefit from protective strategies to reduce the effects of high levels of radiation and temperature during the summer [3,4,5]. Protective netting provides an alternative to the standard practice of evaporative overcooling (EC) to mitigate sunburn in the Pacific Northwest [6,7,8] by reducing fruit surface temperatures [9]. In the last decade, concerns have been raised about increases in water use in agriculture from practices like evaporative cooling. Furthermore, the use of evaporative overcooling (EC) can increase the spread of pathogens such as Sphaeropsis rot (*Sphaeropsis pyriputrescens*) that benefit from elevated humidity for spore release [10]. In WA, the availability of water for irrigated fruit production has not been a limiting factor, and for this reason, EC is still sustainable [5]. However, a study carried out in South Africa reported that EC was less effective than protective netting to reduce sunburn in “Cripps “Pink” and “Royal “Gala” apples [9]. A recent study conducted in New York Stare on “Honeycrisp” confirmed that the netting was the best strategy to reduce sunburn incidence [11].

In some regions, protective netting was initially installed over orchards to mitigate hail damage [12,13]. Subsequently, its expanded uses include protection against environmental stress in areas less affected by hail [14,15,16,17,18,19]. The adoption of protective nets can strongly affect the orchard environment, including reductions in air, leaf, and fruit temperature, and is dependent on the type of net, color, and shading factor [2,3,20]. The primary reasons for deploying protective netting include sunburn protection, water conservation, mitigation of wind and hail damage, and the control of biotic pressures such as birds and insects [20,21,22,23,24].

Apple fruit quality is affected by several preharvest factors in the orchard. Protective netting has also been reported to affect fruit quality at harvest and during storage [3,25,26,27,28]. Photoselective nets are manufactured with a specific shading factor (SF) that indicates the percentage of incoming radiation reduced by the net. Factors such as netting structure/installation, material, filament color, weave style, and net porosity contribute to the SF and light spectral manipulation because of changes to solar radiation geometry, path, and scattering [14,15,29,30,31,32,33,34]. Sometimes, the use of protective netting over crops can adversely impact the over color development of the skin, and this has the potential to adversely impact market grade and price for the seller [16,26,35,36].

“Honeycrisp” [37,38,39] is a high-valued bi-color apple that became popular for its unique fruit quality characteristics and palatability [40,41,42,43]. However, developed initially in a cold climate, it is susceptible to sunburn [11,44,45] and bitter pit [38,46]. As production continues to expand, particularly in warmer regions like Washington State, the use of protective netting to minimize losses to sunburn [2] will be necessary for the economic sustainability of this cultivar. Despite numerous studies carried out on other apple cultivars like “Fuji”, “Braeburn”, “Jonagold”, “Elstar” and “Gala” grown under shading nets [16,25,33,47,48,49,50], there is still limited literature available about the effects of netting on “Honeycrisp” physiology, fruit quality and storability [2,4,11,51,52]. This study’s objectives were to (1) characterize the light intensity and light quality under colored nets in a “Honeycrisp” orchard compared to an uncovered control and (2) investigate the effect of colored nets on external and internal fruit quality for “Honeycrisp”.

## 2. Results

### 2.1. Canopy Light Interception

**Figure 1:**
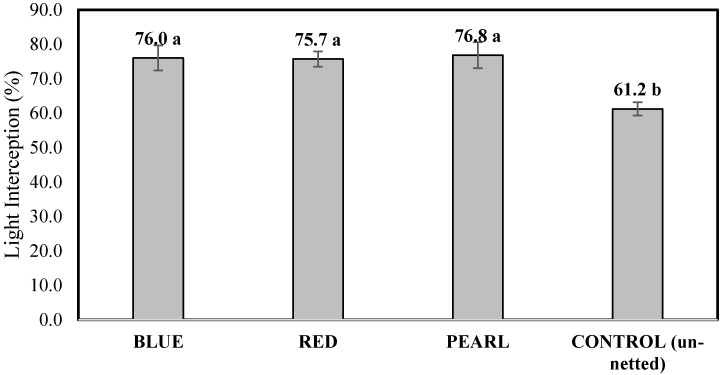
Average light interception (LI) of “Honeycrisp” canopies (%) grown under different photoselective colored nets versus non-netted control across May to July 2016 (average of 3 months, N = 12) in the Quincy area in Washington State. Tukey’s studentized range test was adopted for mean discrimination; values with the same letter indicate non-significance at *p*-value < 0.05. Error bars represent ± standard error.

Canopy light interception under netting was significantly different from the uncovered control only in July 2015, but not in June 2015. Trees under netting intercepted 16 to 22% more light than the uncovered control in July 2015 (Table 1). In 2016, the light interception was not different among different treatments or the uncovered control (Table 1). Additionally, the light interception did not significantly vary during the season in both years. Overall, though, the use of protective netting led to elevated light interception compared to uncovered controls. The light interception was 14.5% to 15.6% lower for uncovered control canopies compared to trees covered with nets when three months were averaged together (Figure 1). However, netting color had no impact on tree light interception across both years of the study.

### 2.2. Spectral Composition of Transmitted and Scattered Light Through Netting

**Figure 2:**
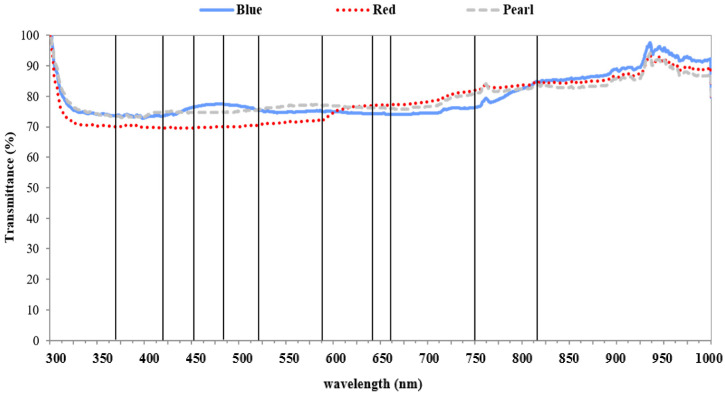
The mean transmittance (%) for spectral wavelengths ranging from 300 to 1000 nm compared to full sunlight (100%, no net) for three colors of netting (N = 4) in 2016. Dark vertical lines over the chart indicate where transmittance values were extracted for statistical analysis among treatments.

Netting treatments showed differences in spectral patterns (300 to 1000 nm) of transmitted light from each specific color of netting (Figure 2). Blue netting transmitted a higher proportion of light from 400 to 550 nm than the red net (Figure 2). Red netting transmitted more light in the spectral range that was just below 600 nm. The pearl net transmitted more light at the lowest wavelengths (UV-vis, 380–750 nm) and transmitted more light, overall, than the other colors of netting (data not shown). Light transmittance was significantly higher for blue netting at 453 nm and 483 nm compared to red netting (Table 2).

**Figure 3:**
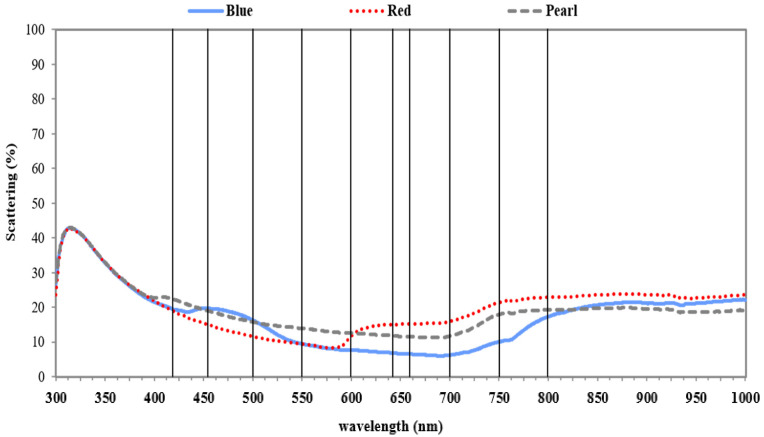
The mean scattered light (%) for spectral wavelengths ranging from 300 to 1000 nm compared to full sunlight for three colors of netting (N = 4) in 2016. Dark vertical lines over the chart indicate where transmittance values were extracted for statistical analysis among treatments.

Light transmittance for pearl netting was not different compared to other colors of netting (Figure 2). The spectra of scattered light under the three colored nets were similar to spectra observed for transmitted light, with scattered light being between 6 and 25% of full sunlight (Figure 3).

For specific spectra, the proportion of scattered light was highest from 453 to 600 nm for pearl and blue (Table 3). However, scattered light was the lowest at these wavelengths for red netting and higher at spectra 600 to 800 nm (Figure 3).

### 2.3. Light Spectra under Colored Nets

All netting treatments reduced the incoming light intensity compared to the uncovered control (Table 4). PAR, UV, blue, red, and far-red light were all significantly lower under netting than the uncovered control (Table 4). The use of nets reduced total PAR (400–700 nm) by approximately 22–25%, and UV light (305–380 nm) decreased by 33% (Table 4). PAR and UV light intensity passing through the nets was unaffected by net color. PAR light in the range of 410–470 nm (blue) was numerically the lowest under red netting; however, no statistical difference emerged between colors. Similarly, red (640–680 nm) and far red (690–750 nm) spectral ranges were the lowest as average under blue netting, but no statistical discrimination between netting color (Table 4). There was more diffuse PAR (213.0 µmol m^−2^ s^−1^) under pearl netting, and it was not statistically different from diffuse PAR in the uncovered control (199.4 µmol m^−2^ s^−1^) nor under the red net (183.4 µmol m^−2^ s^−1^) (Table 4). Similar to transmitted light, scattered light under red netting was the lowest in the blue range (410–470 nm) and well discriminated by means separation (Table 4). There was a 78% increase in red light (640–680 nm) and a 124% increase in far-red light (690–750 nm) under red netting compared to the uncovered control.

Blue/red ratios were significantly different (*p* < 0.001) among the four treatments, and the highest ratios (0.98, similar to uncovered control) were recorded under blue netting in transmitted light. Differences were even more significant among treatments for scattered light, where the blue/red ratio was 2.93 under blue netting. The lowest blue/red ratios occurred under red netting, where ratios were 0.85 and 0.91 for transmitted and diffuse light, respectively. Blue/red ratios for pearl netting were 0.93 and 1.63 for transmitted and scattered light, respectively (Table 4). Red/far-red (R/FR) ratios for transmitted and scattered light were the highest for the uncovered control. Red/far-red (R/FR) ratios for transmitted and scattered light were the lowest under red and pearl netting (Table 4). Although PAR/UV ratios for transmitted light were not dissimilar among treatments, there were differences in ratios for diffuse light. PAR/UV ratios were the highest under pearl netting, followed by red netting and then blue netting (Table 4). The lowest PAR/UV ratios were detected in the uncovered control.

### 2.4. Fruit Quality and Disorder Incidence

**Figure 4:**
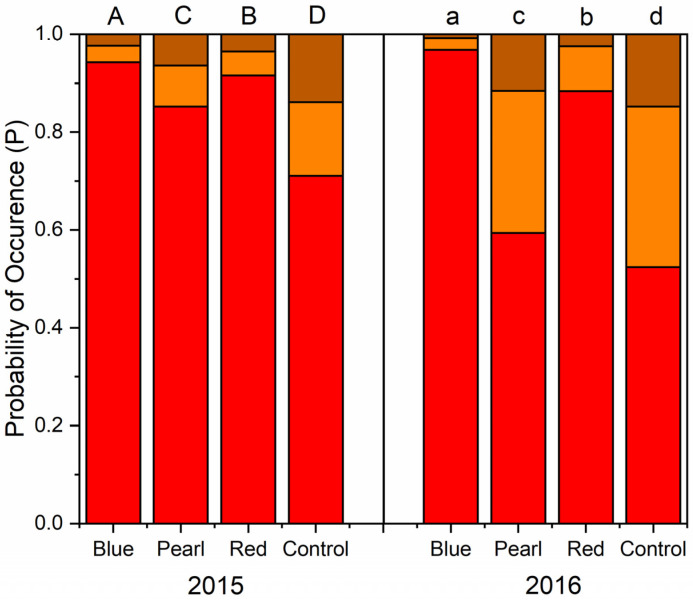
Probability of no or minor sunburn (red), moderate sunburn (orange), or severe sunburn (brown) at harvest for “Honeycrisp” apple under pearl, blue, or red photoselective nets versus an uncovered control in 2015 and 2016. Different letters on top of the columns denote significant differences between treatments in the probability of occurrence of apples with no or minor sunburn symptoms (red).

Productive data collected in 2015 and 2016 did not reveal significant differences between colored net treatments and control non-netted trees for yield/tree, TCSA, and crop load at harvest (Table S1). For both years of the study, more fruit from trees under red and blue netting were unaffected by sunburn compared to pearl and the uncovered control (Figure 4). Fruit from under pearl netting had a higher probability of having no sunburn in 2015 than the control, but there were no differences in 2016 (Figure 4). Fruit only had a 71% and 52% probability of having no sunburn for the uncovered control in 2015 and 2016, respectively. At the same time, fruit from blue or red netting had a 94–97% or 88–92% probability of having no sunburn, respectively. The probability of having no sunburn was lower for pearl netting than the uncovered control but was more significant than either blue or red netting.

**Figure 5:**
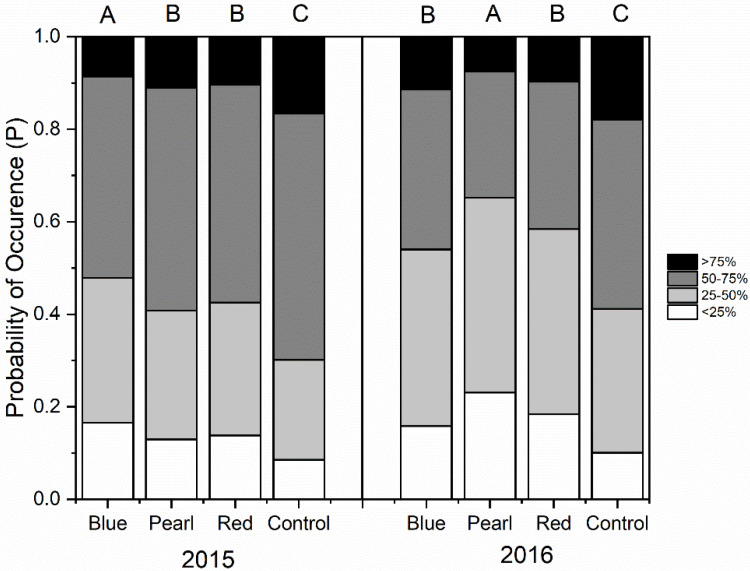
The probability of red overcolor development for “Honeycrisp” apple under pearl, blue, red photoselective nets and an uncovered control in 2015 and 2016. Four categories scale of red covering are: <25% (white), 25–50% (light gray), 50–75% (dark gray), >75% (black). Different letters on top of the columns denote significant differences between treatments in the probability of occurrence of low percentages of red covering (<25%, white).

Similarly, blue and red netting had a lower probability of having moderate or severe sunburn symptoms than pearl netting and much lower than the uncovered control. Apple red overcolor was significantly lower under netting treatments compared to the uncovered control. Fruit under blue netting had the highest probability of having low red overcolor on the fruit. Similar results have been recorded in 2016 (Figure 5).

**Figure 6:**
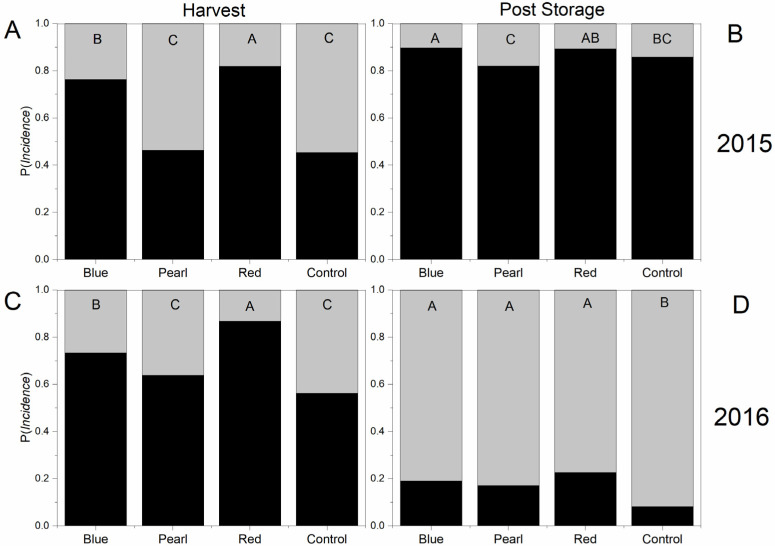
The probability (P) of bitter pit incidence at harvest (**A**: 2015; **C**: 2016) and after four months of storage in the regular atmosphere (**B**; 2015; **D**; 2016) registered for “Honeycrisp” under pearl, blue, red photoselective nets and an uncovered control. Black bars represent the probabilities of no bitter pit incidence on individual fruit, and probabilities of bitter pit incidence are represented by gray bars. Different letters on top of each chart denote significant differences between treatments in the probability of having apples without a bitter pit.

Differences in bitter pit incidence among treatments were similar for both years at harvest. Fruit from under pearl netting and the uncovered control had significantly higher bitter pit incidence. Fruit from under red netting had the lowest incidence of a bitter pit at harvest for both years (Figure 6A,C). In 2015, bitter pit incidence did not increase during storage (Figure 6B) like in 2016 (Figure 6D). Fruit from the uncovered control did not have higher bitter pit incidence after storage in 2015 but was higher in 2016. In 2015, pearl netting had a higher probability of bitter pit incidence after storage compared to the other two netting treatments. However, in 2016, all three netting treatments had a lower probability of bitter pit than the uncovered control (Figure 6).

### 2.5. Fruit Quality Quantitative Characteristics

In 2015, fruit weight and diameter were significantly larger (*p* < 0.05) from under pearl and red netting than fruit from under blue netting or the uncovered control (Table 5). Although in 2016, fruit weight and diameter followed similar trends to 2015, the differences were not statistically significant (*p* > 0.05). Other fruit quality metrics such as firmness, starch index, SSC, and TA were not significantly different among treatments in 2015. Firmness and starch indices did not present significant differences in 2016 either. However, in 2016, SSC was greater for fruit that was uncovered compared to fruit from the three netting treatments. Titratable acidity (TA) was higher in fruit from pearl compared to red netting, and consequently, SSC/TA ratios were higher for fruit from the uncovered control and red netting (Table 5).

## 3. Discussion

### 3.1. Light Manipulation by Use of Photoselective Colored Nets

The reduction of available light and increase in shading can lead to higher vigor and growth, which can alter yield and reduce fruit overcolor [3,20]. In this study, light interception across two seasons was numerically greater in the netted trees but was not always significant (Table 1 and Figure 1). In general, for 2016, trees under netting, regardless of the color of the net, had a 14.5% to 15.6% increase in light interception that reflected the higher vigor of the trees grown under the protective condition in comparison to uncovered trees. While some studies report an optimal range for LI in apple orchards as 60–80%, Robinson et al. [53] recommended 70–75% to reach the best production and tree management [54,55,56]. Light interception rates observed in this study were within this optimum range. Netting can be used as a tool to modify both the quality and quantity of incoming light. Here, different colors of netting changed the transmitted spectra of light reaching the orchard canopy and fruit. Similar to the differences among treatments in this study, Shahak et al. [14] reported that blue netting had higher transmittance at 483 nm. The red netting used in this study had elevated transmittance at just under 600 nm, which was also reported in other studies [15,57,58]. Even with different shading factors, the transmittance for pearl netting was similar to those reported previously by Kong et al. [59]. Here, the pearl net had elevated scattered light spectra in the ranges 450–600 nm and 500–750 nm (Figure 3). Pearl net has already been reported to scatter more light, particularly in the UV region [14,15,59,60,61]. Increased scattered light under pearl netting has been reported to improve light penetration into more shaded portions of the canopies [61]. Diffuse light can improve photosynthesis in late summer when tree canopies are more robust and light penetration more variable [62,63,64].

In the PAR range (400–700 nm), all three netting treatments reduced incoming PAR by approximately 20% [2]. Reductions in PAR, particularly in regions with high solar radiation, led to trees less prone to photo-oxidative stress [5,29,65]. UV radiation decreased similarly for all three netting treatments. UV light was reduced by approximately 33% and was similar to values reported by McCaskill et al. [49] for UV-B radiation. PAR/UV ratios were only different among the four treatments when measured in scattered light. Pearl netting had the highest PAR/UV ratios, and uncovered controls registered the lowest. These ratios were aligned to those reported previously in other regions or other crops [14,60,61]. The alteration of UV light under netting for the whole growing season was reported to affect the antioxidant activity and total polyphenols accumulation in kiwifruit at harvest [60]. Higher PAR/UV was associated with the lower total polyphenol concentration in kiwifruit from under netting [60]. These results suggest that reductions in UV light measured in this study could have an impact on fruit antioxidant concentrations, which can affect overall fruit quality.

In this study, red/far red ratios (R/FR) were lower under netting, with significant differences among netting treatments for both total light and scattered light conditions (Table 4). Unsurprisingly, blue netting had lower R/FR ratios than the uncovered control as reported by Shahak et al. [14] for scattered light. R/FR ratios measured here fell between the values reported by Baraldi et al. [66] for peach trees measured either at the beginning or at the end of the growing season. R/FR ratios contribute to the transition of phytochromes (PHYA and PHYB) between activated P_FR_/inactivated P_R_ states [67]. Phytochromes regulate several plant physiological responses such as germination, dormancy release, flowering, canopy development, photosynthesis and leaf morphology, nutrition, adjacent trees competition, root elongation, responses to abiotic/biotic stress, and fruit quality [68,69]. R/FR ratio has been adopted as a metric to understand the PHY and light-sensing responses [70,71]. Higher far-red light is typical of shaded areas of the canopy or dense vegetation environments with low R/FR ratio that signal to PHYs inducing stem or shoot elongation (auxins involved) and increasing petiole length and other shade avoidance strategies [69,72,73]. Red light can promote meristematic growth compared to the far-red light, and therefore the R/FR ratio can be important for dormancy release and flower bud differentiation [71]. Baraldi et al. [66] reported an effect of low R/FR on shoot elongation, the thickness of the leaf, and the inhibition of branching in peach trees. Later, Leduc et al. [74] revealed that inhibition of branching and stronger apical dominance occurred in low R/FR conditions. The combination of low blue light and low R/FR ratio has been reported in Arabidopsis to induce shoot elongation [73], and these responses correspond with observations made in this study under red netting.

Although the ranges in R/FR ratios were narrow among the netting treatments, blue/red ratio were greater among treatments with ratios under red netting being the lowest, followed by pearl netting and then blue netting had the highest ratios. The increase in blue light under blue netting can induce several physiological responses such as: dwarfing [14,15,60,75], reduced branching [57], decrease in shoot elongation [76], thinner leaves [76], higher photosynthesis [77] decrease in photoinhibition and better light use efficiency [4]. Cryptochromes (CRY) and phototropins (PHiO) are specific photoreceptors for blue and UV light with fundamental roles in several plant responses involved in phototropism, such as bending toward the sun heliotropism and all the plant directional responses [61,71,78]. In this study, trees under the nets developed better canopies (higher LI%) than uncovered trees, and we attribute this primarily to the impact of the nets on reducing tree stress [2,3,4] developed better canopies respect to uncovered trees.

### 3.2. Effect of Photoselective Colored Netting on Disorder Incidence and Apple Fruit Quality

Several studies have reported the effect of the application of netting on fruit quality [25,26,27,48]. One of the most widely reported effects of netting for apples is sunburn incidence. For this reason, crop protection against high radiation and temperature has become one of the main motivations for netting use in regions of the world with high radiation and low hail-risk [2,9,16,79]. Gindaba and Wand [9] compared the responses of “Cripps Pink” and “Royal Gala” apples to the use of shade nets, kaolin application, and evaporative overcooling and reported the most significant reduction of sunburn under 20% black shade nets. Reig et al. [11] reported a reduction in sunburn incidence (−15.5%) on “Honeycrisp” apples using clear polyethylene netting (20% shading factor) in comparison to non-netted control trees in New York. In our study, trees are grown under red and blue netting produced a higher proportion of fruit that was free of sunburn. Ultraviolet and visible light ranges are critical factors affecting sunburn incidence. In our study, all three colored nets reported statistically similar values for PAR (400–700 nm) and UV (305–380 nm) in the orchard trial in the total light measurement setting (Table 4), so it was not possible to confer any benefit to a specific light modification environment in relation to sunburn. Fruit surface temperature is a function of UV-A, B, C intensities, and light intensity and presents another critical factor contributing to sunburn development [8,80,81]. McCaskill et al. [49], indeed, stated that sunburn damage to fruit most often occurs during the afternoon. Here, light measurements were made before solar noon, and therefore, may differ from later in the afternoon when sunburn risk was greater. As previously reported for other cultivars [25,82,83], netting reduced bitter pit incidence for “Honeycrisp” apple (Figure 6). Interestingly, red netting resulted in the lowest bitter pit incidence for both years at harvest and after storage. The blue net also reduced bitter pit incidence. Candian et al. [24] reported a decrease in bitter incidence under pearl net for “Galaval*” cv, but not for “Brookfield^®^” Baigent. These differences could not be accounted for differences among treatments in overall yield or crop load (Table S1).

The red color development is one of the primary factors affecting fruit quality. Low development of red over color under netting can be a limitation on netting by apple producers. The anthocyanins production, responsible for apples’ blush red color, is sensitive to both temperature and light [20,84]. UV-B (280–320 nm) was also a key player in the anthocyanin synthesis in apple [85]. Brklača et al. [26] reported a reduction of red coloration in “Cripps Pink” apple grown under colored nets compared to uncovered fruit. In that study, yellow netting negatively affected red overcolor, while red and white netting showed a less negative effect. Solomakin and Blanke [86] reported that increasing R/FR ratio (i.e., by the use of reflective mulches) led to an increase of light-dependent PAL (phenylalanine ammonia-lyase), which starts the biosynthesis of anthocyanins (phenylpropanoid metabolic pathway) in apple skin; since the nets reduced the R/FR ratio respect to non-netted condition, the red coloration was therefore lower. Solomakin and Blanke [48] reported in “Pinova” and “Fuji” a better red overcolor in the uncovered control compared to netting. Red color development was not consistent among netting treatments between years. In 2015, red color development was lower for fruit under blue netting, but in 2016, red color development was the lowest for fruit under pearl netting. The best coloration was found in the uncovered control in both years. Iglesias and Alegre [16] reported that the darkest netting over “Mondial Gala” for four consecutive years affected red color development for fruit with higher proportions of less colored apples under black net compared to a transparent net and the uncovered control. Treder et al. [87] indicated that darker netting could inhibit red coloration in “Rubinstar” (Jonagold) and “Sampion” apple. Ubi [88] reported that lower carbohydrate availability was associated with lower red color development in apples. Therefore, conditions where netting reduces net photosynthetic rates can also impact the pigmentation of fruit. However, in another report from this study, Mupambi et al. [4] reported no changes in net carbon fixation under netting in high light, semi-arid environments. Do Amarante et al. [25] reported lower red to overcolor development and slower chlorophyll degradation of the peel, a key indicator of fruit maturity [28]. Fruit size is another fruit quality metric considered by the consumer before purchasing [89]. However, consumer preferences for apples differ worldwide and depend on cultural factors, gender, status, consumer age, type of market, and past fruit eating experiences [28]. In the current study, “Honeycrisp” fruit was significantly larger under netting in 2015; this trend was the same in 2016, but not significant.

Similarly, Iglesias and Alegre [16] also reported a positive effect of crystal net on the proportion of higher size (> 80 mm) fruit for “Mondial Gala” apples, but only in the first growing season of the study. An amplified far-red portion of light (lower R/FR ratio) can increase dry matter partitioning [90]. Corollaro et al. [91] reported that “Fuji” apples grown under a red net were characterized by larger cells with higher intercellular space. Bastias et al. [77] also reported an increase in fruit weight of “Fuji” apples grown under blue or gray nets (40% SF) as compared to a white net (20% SF) used as control. In 2015, fruits were the largest under pearl netting and the smallest under blue netting and the uncovered control (Table 5). Furthermore, other fruit quality parameters were not consistently different during the two years of the experiment (i.e., firmness and starch index). This result was consistent with other studies on the effects of netting on apple quality for “Fuji” [16,25], but not for “Gala” [87]. Statistical differences between control and netting in SSC were observed in 2016 but were below 1° Brix, essentially rendering them indistinguishable to the consumer [92]. Decreases in SSC under netting conditions were consistent with previous studies using “Fuji”, “Pinova” [48] and “Gala” [25]. SSC/TA ratios were minimally different among treatments indicating that netting did not affect the flavor of the fruit. Similar results were reported for other studies where titratable acidity was not consistently different among treatments or across years [47,83,93]. SSC/TA was used by Solomakin and Blanke [48] as an indicator of consumer taste in the absence of real sensory analysis, and no differences among netting treatments were reported.

## 4. Materials and Methods

### 4.1. Experimental Sites, Plant Material, and Colored Shading Nets

This study was conducted near Quincy, WA (USA), an area characterized by a desert climate (BSk) according to the Köppen climate classification system [94]. The orchard soil was analyzed for texture and classified as silt loam with 64% silt, 26% sand, and 10% clay; it also had 1% organic matter, electrical conductivity was 0.12 dS/m, and pH (1:1) was equal to 4.87. “Cameron Select^®^ Honeycrisp” on Bud-9 rootstock trees were planted in 2013 with a spacing of 0.6 m × 4.0 m with a planting density of 4485 trees ha^−1^ and trained on 4-wire V-trellis that was oriented north–south.

In spring 2015, pearl, blue and red polyethylene (HDPE) nets (Leno 3640, Polysack Plastics Industries, Negev, Israel) were installed over the top of the orchard (4 m above the ground). Another section of the same orchard was as an uncovered control with no overhead cooling applied. The net shading factor (SF) indicated by the manufacturer ranged from 19 to 23% for the different colors, and we verified the accuracy of this factor ourselves (Figure S2). Net mesh dimensions were 5.2 mm × 2.1 mm, with a strand diameter of 0.3 mm. For each net-covered section in the orchard (made of 4 adjacent orchard rows), the two outside rows served as guard rows. Data were only collected within the two interior rows. Ten replications for each netting color and five replications for the uncovered control were used in 2015, and 12 replications for each netting color and six replications for the uncovered control were used in 2016.

Each year, the netting was deployed after bloom and retracted after harvest. Standard commercial orchard management practices (pruning, fertilization, crop load management, and pesticide application) were applied equally to all trees. Trees were irrigated in the row by drip, and micro-sprinklers were used to maintain the grass strip between rows. For 2015 and 2016, crop load was adjusted less than 30 days after bloom by hand thinning to four fruit cm^−2^ trunk cross-sectional area (TCSA). Yield per tree was recorded for each tree in the trial, and crop load at harvest was calculated as the number of fruit/TCSA cm^2^ (Table S1).

### 4.2. Canopy Light Interception Measurements in the Orchard

Canopy light interception (LI) measurements were made 2 hours before solar noon [95] using a 1 m-long light bar LI-191R Line Quantum Sensor (Li-Cor Biotechnology, Lincoln, NE, USA) and a Li-1500 light sensor logger to record data (Li-Cor Biotechnology, Lincoln, NE, USA). In 2015, measurements were performed in June and July at midday, and in 2016, measurements were made in May, June, and July. Four replications of three adjacent trees with the central tree facing west were used for LI measurements. The light bar was placed 40 cm from the central tree trunk on the ground. LI was calculated from the mean of three light readings from the same position (PAR _transmitted through canopy_). Photosynthetically active radiation (PAR) was measured between rows using a Q53292 Quantum PAR sensor (Li-Cor Biotechnology, Lincoln, NE, USA) and was used as the reference value (PAR _reference_). Canopy light interception (%) was calculated using the following equation:

Light interception (%) = (1 − (PAR _transmitted through canopy_/PAR _reference_)) × 100% (1)

### 4.3. Light Spectral Composition under Netting

Spectral composition of direct light and diffuse light were measured once a month on a full sunny day using a PS-300 spectroradiometer (Apogee Instruments, Logan, UT, USA) for each treatment starting three hours before solar noon every month from May to September 2016 (August measures were affected by clouds that caused too much variability in the measures, for this reason, this month was excluded by the dataset). The PS-300 detector (Apogee Instruments, Logan, UT, USA) was mounted on a tripod 200 cm above ground level and oriented perpendicular to the direction of the sun. The PS-300 spectroradiometer spectral range was 300 nm to 1000 nm measured at 0.5 nm intervals. The spectral composition for full sunlight was used as the reference for calculating transmittance for each treatment. The spectroradiometer was used in “scope mode” and with a detection integration time of 50 ms, five scans were used for each measurement, temperature compensation was “ON”, and Xtiming resolution control was “low”. Two positions under each netting treatment (northern and southern) were selected and marked so that measurements were made in the same place each month. The instrument was placed at the center of each row and oriented to face the sun. Transmittance (expressed as total light under each net / total light outside the net) was measured three times for each netting treatment.

“Radiometer mode” for a PS-100 spectroradiometer (Apogee Instruments, Logan, UT, USA) was used to quantify light scattering, and changes to the spectral composition during scattering were calculated. Total irradiance (micromoles per square meter per second) was measured for each treatment. Diffuse light, which is defined as total light minus the direct sunlight, was then measured using a 4.5 cm-light diffuser (round opaque and black) to block the direct light at 30 cm from the detector and parallel to it in order to shadow it [14,15,59]. Measurements were performed in the same locations as transmittance measurements noted previously.

The monthly transmittance data were averaged for netting treatment to create the transmittance chart. Specific wavelengths along the full spectrum (300 nm–1000 nm; included four wavelengths related to the absorption peaks of Chl a and Chl b, respectively 420 nm, 660 nm, and 453 nm and 643 nm; [96]) were selected to compare transmittance and scattering of each netting treatment to identify differences at physiologically relevant light spectra wavelengths. Light intensity parameters (PAR, UV (UV-A+UV-B), blue, red, far-red) were expressed as µmol m^−2^ s^−1^ were calculated in the same ranges as Kong et al. [59] and light quality ratios (blue/red, red/far red and PAR/UV) presented [15].

### 4.4. Fruit Quality Evaluation

Harvest occurred on the 26 August and the 24 August in 2015 and 2016, respectively. 16 fruit per tree or less when crop load was smaller were collected from ten trees per replication in 2015 and five trees per replication in 2016. Half of the harvested fruits were conditioned at 10 °C for 7 days and then stored in a regular atmosphere at 2 °C for four months. The remaining half was analyzed for external and internal quality parameters immediately following harvest. Diameter (mm) and weight (g) of each fruit were measured, and the means per replication were used for data analysis. Sunburn severity was evaluated only at harvest using a modified six-level scale [2] where fruits from “0” and “1” categories were assigned to the clean or minor sunburn category, fruit belonging to “2” was assigned to the moderate sunburn category, and fruit belonging to “3” and “4” was assigned to the severe sunburn category, and “5” describes sunburn necrosis (no apples found under this category in the present study). The red color coverage was visually evaluated only at harvest using a four-level scale that classified apples in 0 to 25%, 25 to 50%, 50 to 75%, and 75 to 100% of red coverage. Bitter pit presence was noted for each fruit, and total bitter pit incidence was recorded for each replicate [46]. Firmness was measured and averaged from the sun and shade side of each apple fruit using a fruit texture analyzer (Güss^®^, GS 20, Strand, South Africa) with an 11 mm metal probe. The starch index was determined from the bottom half of each apple after sprayed with Lugol’s solution (15 g·L^−1^ potassium iodine and 6 g·L^−1^ elemental iodine) using a hand-held spray bottle and left for 10 minutes. Starch content was then rated on a scale from 1 (more starch present) to 6 (no starch present). Soluble solids content (SSC, expressed in °Brix) was measured by using a transverse section of each fruit and squeezing approximately 1 mL of juice onto a digital hand-held refractometer (ATAGO^®^, Model PAL-1, Tokyo, Japan). Titratable acidity (TA) was determined by titrating 5 mL of juice with 1 M NaOH with an automated titrator (Model 719 S, Metrohm AG, Hersiau, Switzerland); the values were expressed as a percentage of malic acid.

### 4.5. Statistical Analysis

Light interception, transmittance, scattering light, light intensity and quality were analyzed with PROC GLM (general linear models) in SAS (SAS Institute Inc., Cary, NC, USA) using type III SS and Tukey’s studentized range post hoc test to discriminate among means (α = 0.05). Fruit quality continuous variables like SSC, TA, firmness, fruit weight, and diameter were analyzed with PROC GLM in SAS using type III SS and a Tukey post hoc test to discriminate among means. All the tests used a 95% level of confidence. Using the proportional odds model, variables with an ordinal response (scales with an intrinsic order) like color coverage and sunburn were analyzed with PROC LOGISTIC in SAS. Variables with a nominal response (scales with no intrinsic order) like bitter pit incidence and background color were analyzed with PROC LOGISTIC in SAS, using the generalized logit function. The proportional odds model compares the probability of each treatment being in lower categories of the evaluated scale, while the generalized logit function compares the odds of each treatment of being in a specific category [97,98,99].

## 5. Conclusions

Netting can be used to manipulate light radiation reaching the canopy and fruit surface. Netting can affect total incoming radiation and the spectral composition, which can have implications for tree growth, productivity, and overall quality. Here, the use of blue, red, and pearl netting all increased total light interception because of more robust canopies with more vegetative growth and leaf area. Netting treatments also changed the composition of visible light as well as the ratios of visible/UV light. Here, netting reduced both the incidence of sunburn and bitter pit in “Honeycrisp” apples for two years. Blue netting was the most effective at controlling sunburn but also reduced red color development on the fruit surface. Generally, all netting treatments reduced red color development compared to an uncovered control. Blue and red netting consistently reduced bitter pit incidence in fruit compared to the uncovered control in the present experimental condition. Other than small reductions in red color development that may be associated with differences in fruit maturity, “Honeycrisp” apple quality was not considerably affected by netting treatments (Table S3). Fruit weight was greater in the first year under netting, and similar trends were also observed in the second year of the study. Further years of evaluations could be beneficial to highlight and confirm similar trends under the nets. To enhance future netting adoption, there is a need to minimize red color development reductions and better understand differences in maturity under netting. Furthermore, management strategies to mitigate reductions in fruit color, such as changing netting color, retracting netting during harvest, or deploying additional reflective fabric in the inter-rows, will be important to investigate in the future. With careful management, the use of colored nets can help to reduce losses to physiological disorders like sunburn and bitter pit in “Honeycrisp” apple.

## Figures and Tables

**Table 1:** The effects of colored nets (blue, red, pearl) vs. non-netted open-field control on light interception (LI%) of “Cameron Select^®^ Honeycrisp” trees (3rd and 4th leaf) grown in the Quincy area in Washington State in May, June and July 2015 and May, June and July 2016. Means are expressed as $\bar{x}$ ± standard error (N = 4). NS, ** indicate, respectively no significance or significance at *p*-values of <0.01. Tukey’s studentized range test was adopted for mean discrimination, and in columns, means with the same letter indicate non-significance at *p*-value <0.05. The column “significance by month” reports the significance of the comparison between the two or three months for each netting treatment (net color).

Year	Net Color	Canopy Light Interception (%)			Significance by Month
		May	June	July	
2015	BLUE		77.6 ± 2.1	76.2 ± 0.6 A	NS
	RED		75.2 ± 3.9	81.2 ± 1.2 A	NS
	PEARL		79.4 ± 5.3	75.8 ± 3.9 A	NS
	CONTROL		70.6 ± 8.9	59.2 ± 5.3 B	NS
	Significance		NS	**	
2016	BLUE	75.2 ± 8.0	74.5 ± 6.1	78.4 ± 6.6	NS
	RED	75.0 ± 6.4	75.4 ± 2.5	76.6 ± 2.6	NS
	PEARL	74.7 ± 6.6	79.1 ± 6.3	76.5 ± 8.3	NS
	CONTROL	59.6 ± 3.5	60.8 ± 4.3	63.1 ± 2.8	NS
	Significance	NS	NS	NS	

**Table 2:** Mean transmittance (%) in 2016 for blue, pearl, and red netting at 370, 420, 453, 483, 519, 589, 643, 660, 750, and 817 nm (N = 4). Letters in each column denote significance among netting treatments determined using Tukey’s studentized range test (α = 0.05). * *p* < 0.05, NS = not significant.

Transmittance% at λ (nm) Across 4 Months												
Net Color	370 nm	420 nm	453 nm		483 nm		519 nm	589 nm	643 nm	660 nm	750 nm	817 nm
Blue	73.7	73.5	76.5	A	77.5	A	75.4	74.9	74.3	74.1	76.3	84.9
Pearl	73.4	74.8	74.7	AB	74.9	AB	75.4	77.0	76.2	75.9	80.7	83.8
Red	70.0	69.6	69.7	B	70.1	B	70.4	72.2	77.0	77.1	81.9	84.7
*Significance of net color*	*NS*	*NS*	*		*		*NS*	*NS*	*NS*	*NS*	*NS*	*NS*

**Table 3:** Mean scattered light (%) in 2016 for blue, pearl, and red netting at 420, 453, 500, 550, 600, 643, 660, 700, 750 and 800 nm (N = 4). Letters denote significance among netting treatments determined using a Tukey’s studentized range test (α = 0.05). ** = *p*<0.01, *** = *p* < 0.001, NS = not significant within each column.

Scattering% at λ (nm) across 4 Months										
Net Color	420 nm	453 nm	500 nm	550 nm	600 nm	643 nm	660 nm	700 nm	750 nm	800 nm
Blue	19.3	19.7 A	16.2 A	9.4 B	7.6 B	6.8 C	6.5 C	6.2 C	10.0 C	17.3 C
Pearl	22.2	19.1 A	15.7 A	13.9 A	12.5 A	11.8 B	11.5 B	11.7 B	17.9 B	19.2 B
Red	18.7	15.1 B	11.6 B	9.3 B	11.7 A	15.1 A	15.2 A	15.9 A	21.4 A	22.9 A
Significance	NS	**	**	***	***	***	***	***	***	***

**Table 4:** Total PAR (photosynthetic active radiation, µmol m^−2^ s^−1^) and spectral ratios of transmitted (top) and scattered (bottom) light under pearl, blue, and red netting compared to an uncovered control in “Honeycrisp” apple in 2016. Significance: *** *p* < 0.001, NS = not significant. Significance was established with proc GLM (general linear models) in SAS, type III SS, and Tukey’s studentized range as a post hoc test to discriminate means. Same capital letters within each column mean no difference between treatments.

Quincy Commercial Block (2016)			Light Intensity (µmol m^−2^ s^−1^)										Light Quality (ratios)					
Light Type	Color Nets-ctrl	N	PAR: 400–700 nm		UV: 305–380 nm		Blue: 410–470 nm		Red: 640–680 nm		Far Red: 690–750 nm		Blue/Red		Red/Far Red		PAR/UV	
Total full light (transmitted)	BLUE	48	1407.1	B	63.1	B	217.2	B	222.5	B	303.7	B	0.98	A	0.73	B	22.65	
	RED	47	1405.3	B	60.9	B	203.5	B	240.7	B	335.3	B	0.85	C	0.72	C	23.45	
	PEARL	48	1470.4	B	64.5	B	220.9	B	237.1	B	329.7	B	0.93	B	0.72	C	23.09	
	Open field non-netted ctrl	4	1888.1	A	90.0	A	292.6	A	301.7	A	407.7	A	0.97	A	0.74	A	21.33	
Significance			***		***		***		***		***		***		***		NS	
Scattered light (diffuse)	BLUE	48	159.5	B	20.4	B	42.5	B	14.5	D	23.0	C	2.93	A	0.63	B	7.89	C
	RED	48	183.4	AB	19.6	B	32.8	C	36.1	A	59.5	A	0.91	D	0.60	BC	9.47	B
	PEARL	48	213.0	A	21.0	B	44.7	AB	27.5	B	46.5	B	1.63	C	0.59	C	10.21	A
	Open field non-netted ctrl	4	199.4	A	31.5	A	51.7	A	20.2	C	26.6	C	2.55	B	0.76	A	6.44	D
Significance			***		***		***		***		***		***		***		***	

**Table 5:** Quantitative “Honeycrisp” apple quality instrumental parameters of fruit picked under pearl, blue, and red netting compared to apples from non-netted control trees in the Quincy commercial block (WA) in 2015 and 2016. Data from “at harvest” and 4-month postharvest apples were combined. Significance: ** *p* < 0.01, NS = non-significant. The same letter means no significant difference according to Tukey’s test within each column. (N = number of apples evaluated for each treatment).

	Color Nets	N	Fruit (N=)	Diameter (mm)	Weight (g)	Firmness (kg/cm^2^)	Starch Index	SSC (° Brix)	TA (% Malic Acid)	SSC/TA Ratio
2015	Control	6	380	83.3 B	232 B	15.4	4.8	14.9	0.71	20.9
	Blue	12	703	84.9 B	245 B	15.1	5.2	14.6	0.70	20.9
	Pearl	12	709	88.5 A	275 A	15.2	5.1	14.8	0.70	21.1
	Red	12	745	86.9 AB	259 AB	15.1	4.9	14.8	0.70	21.1
	Significance			**	**	NS	NS	NS	NS	NS
2016	Control	6	144	97.9	385	14.5	4.1	15.2 A	0.69 AB	22.0 A
	Blue	12	315	98.8	397	13.9	4.8	14.4 B	0.70 AB	20.8 AB
	Pearl	12	320	100.2	397	14.4	4.2	14.6 B	0.73 A	20.1 B
	Red	12	349	98.1	387	14.1	4.5	14.4 B	0.66 B	21.7 A
	Significance			NS	NS	NS	NS	**	**	**

## Supplementary Materials

The following are available online at https://www.mdpi.com/2223-7747/9/12/1708/s1, Table S1: Yield (kg), trunk cross-sectional area (TCSA) (cm^2^), and crop load (fruit cm^−2^ TCSA) (±SEM) for trees under blue, red, or pearl netting compared to an uncovered control (Control) in 2015 and 2016. Letters denote differences determined using a post hoc Tukey’s test (α = 0.05). Figure S1: Shading factor (SF, %) by the three colored nets used in this trial on “Honeycrisp” in Quincy (WA) in 2015–2016. Average values of shading factor were obtained by the measurements of transmittance from the full measured spectrum (300 nm to 1000 nm) and in the range 400 nm to 700 nm (PAR). Shading factor (%) is calculated following the formula shading factor (%) = 100% light– transmittance (%) equal to the portion of light filtered by the nets. Measures were taken in the same locations in the block month after month from May to September 2016 (N = 4, no August). NS is indicating no statistical difference (*p* > 0.05). Table S2: Effect of photoselective colored nets in modifying the light underneath and summary of physiological responses variation under nets (trees or fruit) based on parameters measured in this study in the Quincy commercial block (WA) for “Honeycrisp” in 2015–2016. Letters populating the last 4 columns on the right come from the statistical analysis mean discriminations reported in the results, Section 3, (same letters mean no difference between the four treatments, and each line is an independent statistical analysis). Comparisons are allowed only along each row, not between rows.

## Abbreviations

PAR	photosynthetic active radiation
UV	ultraviolet
LI	light interception
EC	evaporative overcooling
SF	shading factor
avr	average
BP	bitter pit
SSC	soluble solids content
TA	titratable acidity
