# Supplementary material for: Photoselective Protective Netting Improves “Honeycrisp” Fruit Quality
Source: Plants (Basel). 2020 Dec 4;9(12):1708. doi: 10.3390/plants9121708 (PMC7761960; doi:10.3390/plants9121708)
Supplement: Supplementary file 1 [file plants-09-01708-s001.pdf]

Table S1: Yield (kg), trunk cross-sectional area (TCSA) (cm^2), and crop load (fruit cm^{-2} TCSA) (± SEM) for trees under blue, red, or pearl netting compared to an uncovered control (Control) in 2015 and 2016. Letters denote differences determined using a post hoc Tukey's test ($\alpha = 0.05$).

Treatment	Yield (kg)	TCSA (cm^2)	Crop Load (Fruit cm^{-2} TCSA)
		**2015**	
Blue	4.01 ± 0.17 a	3.93 ± 0.04 a	4.94 ± 0.48 a
Red	4.30 ± 0.61 a	4.01 ± 0.03 a	4.66 ± 0.27 a
Pearl	4.54 ± 0.38 a	4.02 ± 0.07 a	5.09 ± 0.37 a
Control	5.29 ± 1.27 a	4.03 ± 0.05 a	5.51 ± 0.78 a
p-Value	0.65	0.50	0.77

Treatment	Yield (kg)	TCSA (cm^2)	Crop Load (Fruit cm^{-2} TCSA)
		**2016**	
Blue	2.79 ± 0.61 a	5.14 ± 0.54 a	1.34 ± 0.32 a
Red	5.22 ± 0.96 a	5.20 ± 0.42 a	2.42 ± 0.45 a
Pearl	5.09 ± 0.50 a	5.46 ± 0.36 a	2.27 ± 0.21 a
Control	3.22 ± 0.91 a	4.68 ± 0.17 a	1.72 ± 0.49 a
p-Value	0.07	0.57	0.18

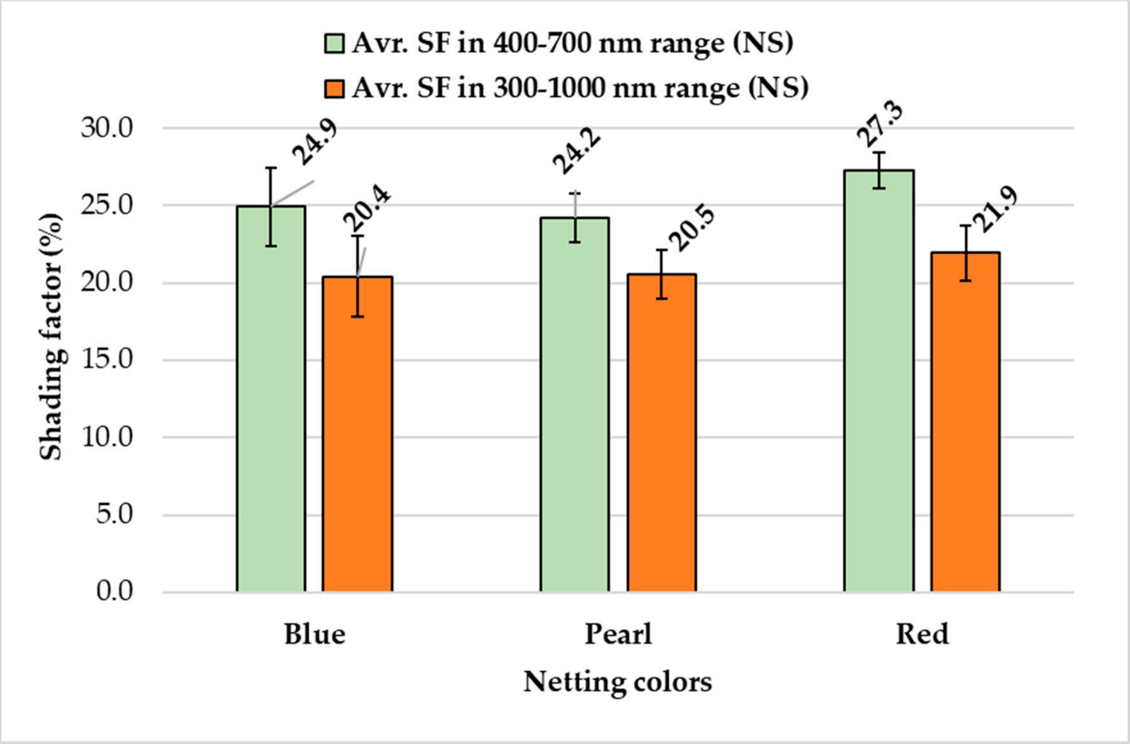

Figure S1: Shading factor (SF, %) by the three colored nets used in this trial in a Honeycrisp in Quincy (WA) in 2015-2016. Average values of shading factor were obtained by the measurements of transmittance from the full measured spectrum (300 nm to 1000 nm) and in the range 400 nm to 700 nm (PAR). Shading factor (%) is calculated following the formula shading factor (%) = 100% light – transmittance (%) equal to the portion of light Figure 2016. (N=4, no August). NS is indicating no statistical difference ($p>0.05$).

Table 2. Effect of photoselective colored nets in modifying the light underneath and summary of physiological responses variation under nets (trees or fruit) based on parameters measured in this study in Quincy commercial block (WA) of 'Honeycrisp' in 2015-2016. Letters populating the last 4 columns on the right come from the statistical analysis mean discriminations reported in the results, Section 3, (same letters means no difference between the four treatments, and each line is an independent statistical analysis). Comparisons are allowed only along each row not between rows.

**Parameters Measured in this Study**	**Year**	**Target of Response**	**Control Un-netted trt (Used as Reference)**	**Pearl Net (response under)**	**Red Net (Response Under)**	**Blue Net (Response Under)**
Shading Factor (300 nm-1000 nm, %)	Avr 4M 2016	net	N/A	20.5 (a)	21.9 (a)	20.4 (a)
PAR (μmol m^{-2} s^{-1})	2016	Total full light under net	a	b	b	b
PAR (μmol m^{-2} s^{-1})	2016	*Scattered light under net*	ab	a	b	b
UV (μmol m^{-2} s^{-1})	2016	Total full light under net	a	b	b	b
UV (μmol m^{-2} s^{-1})	2016	*Scattered light under net*	a	b	b	b
Blue/Red ratio	2016	Total full light under net	b	c	d	a
Blue/Red ratio	2016	*Scattered light under net*	a	b	c	a
Red/ Far red	2016	Total full light under net	a	c	c	b
Red/ Far red	2016	*Scattered light under net*	a	c	c	b
PAR/UV ratio	2016	Total full light under net	a	a	a	a
PAR/UV ratio	2016	*Scattered light under net*	d	a	b	c
Light interception	July 2015	trees	b	a	a	a
Light interception	Avr 3M 2016	trees	b	a	a	a
Sunburn-free/minimal incidence	2015	apples	d	c	b	a
Sunburn-free/minimal incidence	2016	apples	d	c	b	a

Poor Red overcolor (<25%)	2015	apples	c	b	b	a
Poor Red overcolor (<25%)	2016	apples	c	a	b	b
Bitter Pit-free incidence	2015 (harvest)	apples	c	c	a	b
Bitter Pit-free incidence	2016 (harvest)	apples	c	c	a	b
Bitter Pit-free incidence	2015 (4M postharvest)	apples	bc	c	ab	a
Bitter Pit-free incidence	2016 (4M postharvest)	apples	b	a	a	a
Average fruit weight (g)	2015 (harvest)	apples	b	a	ab	b
Average fruit weight (g)	2016 (harvest)	apples	a	a	a	a
Firmness (kg/cm^2)	2015 (harvest)	apples	a	a	a	a
Firmness (kg/cm^2)	2016 (harvest)	apples	a	a	a	a
SSC/TA ratio	2015 (harvest)	apples	a	a	a	a
SSC/TA ratio	2016 (harvest)	apples	a	b	a	ab
